# The metabolic axis of macrophage and immune cell polarization

**DOI:** 10.1242/dmm.034462

**Published:** 2018-07-06

**Authors:** Spencer A. Hobson-Gutierrez, Carlos Carmona-Fontaine

**Affiliations:** Center for Genomics and Systems Biology, Department of Biology, New York University, New York City, NY 10003, USA

**Keywords:** Cancer metabolism, Immunometabolism, Tumor microenvironment

## Abstract

The extracellular space of solid tumors ranges from being well-nurtured to being completely ischemic and can serve as a source of intratumoral heterogeneity, determining the behavior and molecular profiles of malignant and stromal cells. Here, we discuss how the metabolic tumor microenvironment modulates the phenotypes of the immune cells that infiltrate tumors, with an emphasis on tumor-associated macrophages. These cells constitute a diverse population that has pro-tumoral and anti-inflammatory properties, and are likened to anti-inflammatory ‘M2’ macrophages. Recent findings show how different metabolic microenvironments specify an array of phenotypic changes in macrophages. In tumors, extracellular metabolite levels vary predictably according to proximity to the vasculature, and phenotypic changes in tumor-associated macrophages and in other immune cells are also predictable. We speculate that this ‘metabolic axis’ of macrophage polarization modulates – and is modulated by – the response to inflammatory cues, creating a wide variety of possible phenotypic states. Understanding how extracellular metabolites influence cell phenotypes allows us to predict how tumor-associated macrophages and other tumor cells might change, with the aim of harnessing this predictability for therapy. Overall, we describe an emerging picture in which chemokines, growth factors and the metabolic tumor microenvironment act together to determine the phenotypes of tumor-infiltrating immune cells.

## Abstract (precis)

**Summary:** Extracellular metabolites can mediate cell communication and change transcriptional and functional aspects of tumor and normal cells. This Review focuses on how these metabolic cues affect the immune system with a special focus on tumor-associated macrophages.

## Introduction

Interactions between cancer and immune cells play a crucial role in tumor initiation, growth and metastasis. Tumor-associated macrophages (TAMs; see Glossary, Box 1) are particularly abundant within tumors and have been shown to enhance tumor malignancy; the ability to inhibit these pro-tumoral effects could open up potentially new therapeutic avenues (De Palma and Lewis, 2013; Murray, 2017; Noy and Pollard, 2014; Quail and Joyce, 2017). Unfortunately, our understanding of how TAMs acquire the ability to promote tumor progression remains incomplete and the definition of a bona fide TAM population remains controversial (Franklin et al., 2014; Murray et al., 2014; Ostuni et al., 2015; Qian and Pollard, 2010; Ruffell et al., 2012; Sica and Mantovani, 2012; Wei et al., 2013).

**Box 1. Glossary**

**Anergy:** A considerably low immune response to an antigen.

**Cytotoxic T lymphocytes (CTLs):** Effector T cells that specialize in killing cells recognized as being foreign, including bacteria, pathogens and cancer cells, which present mutated proteins that act as antigens.

**Erythro-myeloid progenitors:** A family of undifferentiated blood or bone marrow cells.

**Inflammasome:** A protein complex responsible for the activation of inflammatory responses.

**Kupffer cells:** Tissue-resident macrophages from the liver.

**Lipopolysaccharides (LPSs):** Large molecules found on the outer membrane of gram-negative bacteria that can activate macrophages and other immune cells.

**Microglia:** A type of glia in the central nervous system that shares embryological and functional properties with tissue-resident macrophages from other organs.

**Myeloid-derived suppressor cell:** A type of immune cell from the myeloid lineage that has strong immunosuppressive activities, and that is normally associated with pathological conditions, such as cancer.

**Natural killer (NK) cell:** A white blood cell that releases cytotoxins in response to recognizing dysfunctional or cancerous somatic cells.

**Oncometabolite:** A metabolite that drives malignant transformation in a process akin to that driven by oncogenes.

**Phosphoenolpyruvate:** A metabolic intermediate involved in glycolysis and gluconeogenesis.

**Pseudohypoxia:** A condition in which cells mount a hypoxic response (for example, by increasing HIF-1α levels) under normal oxygen conditions.

**Regulatory T cell (Treg):** An immune cell that commonly acts as an immunosuppressive cell and is involved in autoimmune prevention and self-antigen tolerance.

**T helper cell:** a type of T cell that is particularly important in adaptive immunity. T helper cells express the surface protein CD4 and are also known as CD4^+^ cells. They help the activity of other immune cells by releasing cytokines that suppress or regulate T cell activity, B cell antibody class switching, and increase the bactericidal activity of macrophages. Subtypes of this cell population include Type 1 helper T (T_h_1), Type 2 helper T (T_h_2) and Type 17 helper (T_h_17) cells.

**Reductive carboxylation:** A carboxylation step (production of a carboxylic acid group by adding CO_2_ to a substrate) coupled with a reductive reaction. Here, it refers to the production of alpha-ketoglutarate from exogenous glutamine, which allows cancer cells to use the TCA cycle in reverse to produce citrate and other metabolites.

**Tumor-associated macrophage (TAM):** A type of macrophage that infiltrates solid tumors and that often displays pro-tumoral properties.

The regulation of cell metabolism is now recognized to be a crucial aspect of the immune system (Ganeshan and Chawla, 2014; O'Neill and Hardie, 2013; Olenchock et al., 2017; Pearce et al., 2009). Virtually every aspect of the immune response, including antigen presentation and response (Angelini et al., 2002; Doedens et al., 2013; Rubic et al., 2008), clonal expansion of activated T cells (Chang et al., 2015; Man et al., 2013; Wang et al., 2011), immune cell differentiation (Dang et al., 2011; Pearce et al., 2009; Shi et al., 2011b; Sinclair et al., 2013) and the role of the immune system in wound healing (Barbul et al., 1990; Cattin et al., 2015), has been shown to be intimately linked with cell metabolism. It has also become evident that the immune system has a central role in regulating whole-body metabolism (Buck et al., 2017; Exley et al., 2014; Man et al., 2017; Odegaard and Chawla, 2013). Much of the attention, however, has focused on how different chemokines, and other cell signals, modulate and rewire intracellular metabolic networks (Ganeshan and Chawla, 2014; O'Neill and Hardie, 2013; O'Neill and Pearce, 2016; Pearce et al., 2013).

This Review focuses on how metabolites, especially extracellular metabolites, can act as signals that regulate immune cell function, behavior and phenotype. Although most of our discussion centers on macrophages, we also discuss how the metabolic microenvironment can modulate the phenotype of other immune cells. A key role of immune cells is to sense changes to the environment and to respond when they identify a threat to homeostasis (Buck et al., 2017; Kotas and Medzhitov, 2015; Okabe and Medzhitov, 2016). With that in mind, it is perhaps not surprising that immune cells are particularly sensitive to changes in metabolic conditions during normal physiology and disease, requiring them to integrate signals, such as interleukins, cytokines and chemokines, with cues such as the local oxygen concentrations and other metabolites. Before addressing the sources of macrophage phenotypic variability, we will briefly discuss how the metabolic microenvironment of solid tumors is inescapably heterogeneous.

## Heterogeneity in the metabolic microenvironment of tumors

Tumor cells display remarkable alterations in their metabolism, such as pseudohypoxia (Box 1) (Frezza et al., 2011), reductive carboxylation (Mullen et al., 2012) and the generation of oncometabolites (Box 1). In the best studied of these alterations, the Warburg effect, cancer cells display a highly glycolytic metabolism even in the presence of oxygen (Dang et al., 2009). This metabolic mode is characterized by high rates of lactate production and glucose consumption, which partially compensates for the lower adenosine triphosphate (ATP) yield (per glucose molecule) when we compare this mechanism with the ATP yield of oxidative phosphorylation (Liberti and Locasale, 2016). Warburg saw this effect as a detrimental aberration, and he hypothesized that tumor cells had dysfunctional mitochondria (Koppenol et al., 2011). However, it is now clear that although some tumors might have mitochondrial alterations, for the most part, they are still functional in most cases (Reznik et al., 2017). A new look at this metabolic strategy has revealed that it enables sufficient ATP to be produced, while permitting cells to retain carbon-rich macromolecules with which to biosynthesize essential intermediates and the large quantities of biomass that are required for tumor proliferation (Lunt and Vander Heiden, 2011; Pavlova and Thompson, 2016). For example, oncogenic alteration can lead to the accumulation of phosphoenolpyruvate (Box 1), shunting glycolytic intermediates into anabolic pathways for pyrimidine, glycerol, serine and glycine synthesis (Locasale et al., 2011; Mazurek et al., 2001; Possemato et al., 2011). The complete catabolism of glucose in an entire round of oxidative phosphorylation yields ∼30 molecules of ATP, but there are no carbon atoms left over for the production of biomass. In contrast, glycolysis yields two molecules of ATP per molecule of glucose, but the two resulting three-carbon molecules of lactate can be reused by normal and tumor cells as sources of energy and carbon (Faubert et al., 2017; Hui et al., 2017). Since the discovery of the Warburg effect, it has become increasingly clear that similar metabolic changes also occur in normal, rapidly proliferating cells, such as in stem cell niches (Zhang et al., 2012), during the clonal expansion of T cells (Wang et al., 2011), and in proliferating cells during wound healing (Lee et al., 2009). These data suggest that the Warburg effect, or aerobic glycolysis, is a strategy that is widely used by proliferating cells rather than being a metabolic aberration (Vander Heiden et al., 2009).

**Fig. 1.**
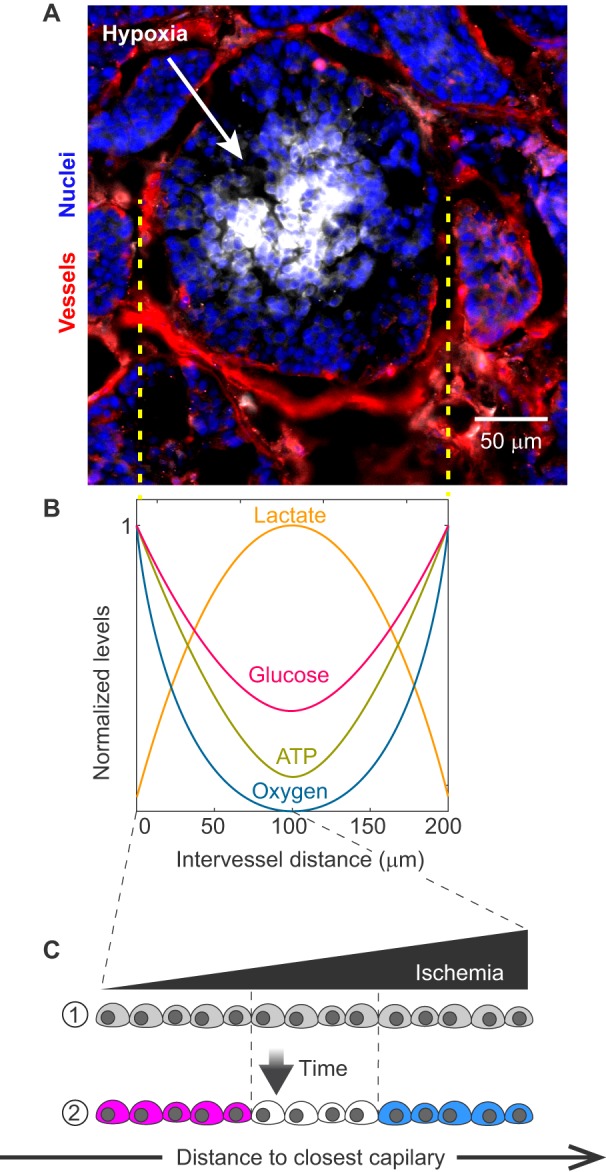
**Metabolic gradients in a tumor.** (A) Immunofluorescence of a breast tumor section obtained from the mouse mammary tumor virus (MMTV-PyMT) model. Blood vessels were stained with antibodies against CD31 (red), hypoxic regions were labeled by detection of pimonidazole (white), and cell nuclei were counterstained with Hoechst (blue). Note how hypoxic regions correlate with distance from the vasculature, with strong hypoxia appearing ∼100 mm from capillaries. (B) Changes in the levels of other metabolites, including lactate and glucose, are also expected within the tumor region defined by the dashed yellow lines. Based on Vaupel (2004). (C) A schematic showing how levels of extracellular metabolites can change with increasing ischemia and with increasing distance from the nearest capillary. Such changes can alter cell phenotypes and provide spatial information to tumor cells.

These cancer-related metabolic alterations, and their consequences in the extracellular space, enhance intratumoral heterogeneity. Rapid cell proliferation in most tumors cannot be matched by revascularization, which results in inefficient blood perfusion and in regions experiencing extensive ischemia (Gatenby and Gillies, 2004; Thomlinson and Gray, 1955). These conditions create large microenvironmental differences between cells that are proximal or distal to the vascular network. Thus, the metabolic microenvironment of the tumor changes along relatively smooth gradients that range from well-perfused perivascular regions to ischemic regions, where lactic acid accumulation and nutrient deprivation lead to necrotic zones (Carmona-Fontaine et al., 2013; Gatenby and Gillies, 2004; Thomlinson and Gray, 1955; Vaupel, 2004). Under conditions of nonlethal ischemia, acidosis, hypoxia and nutrient deprivation, metabolites can act as signals that modulate cellular functions (Fig. 1) (Buck et al., 2017; Carmona-Fontaine et al., 2017; Gatenby and Gillies, 2004). This tumor-induced metabolic diversity suggests that infiltrating macrophages could experience a variety of different microenvironments, but first we need to understand how macrophages alter their phenotype under normal homeostasis.

## Macrophages are a diverse cell population

Before discussing the variability of TAMs and the effect of metabolites on this diversity, we need to briefly address the intrinsic variability of macrophages. Macrophages are an extremely diverse cell population even in homeostasis (Gordon and Taylor, 2005). They infiltrate and reside in virtually all of our organs, and the local microenvironment of these different compartments plays a key role in establishing and maintaining this diversity (Amit et al., 2016; Gosselin et al., 2014; Lavin et al., 2017). Many aspects of specific organs alter macrophage phenotypes, including the tissue microarchitecture (Gonzalez et al., 2013; Mebius and Kraal, 2005), the rate of metabolic activity (Okabe and Medzhitov, 2014; Thorburn et al., 2014), exposure to commensal microorganisms in the case of gut and skin macrophages (Chang et al., 2014; Tamoutounour et al., 2013), and the local repertoire of growth factors and hormones (Gosselin et al., 2014; Lavin et al., 2017). Linked to these regional differences, macrophages that reside in different organs might also have distinct embryological origins. Most, if not all, macrophages present at birth develop from yolk-sac-derived erythro-myeloid progenitors (Box 1) (Gomez Perdiguero et al., 2015), and emerge independently of the hematopoietic process (Hashimoto et al., 2013; Yona et al., 2013). Microglia, Kupffer cells (Box 1) and other tissue-resident macrophages mostly originate from this embryonic pool (Ginhoux et al., 2010; Schulz et al., 2012). In other organs, such as in the gut, the initial embryonic-derived macrophage population is replaced over time by macrophages that are derived from circulating monocytes and that are re-supplied by the bone marrow in the hematopoietic process throughout adult life (Bain et al., 2014; Perdiguero and Geissmann, 2016; Schulz et al., 2012). At a finer scale, changes in the levels and repertoire of receptors (Gordon and Taylor, 2005; Lavin et al., 2017), and in the versatile biosynthetic capacity of macrophages (Artyomov et al., 2016; Ganeshan and Chawla, 2014), further add to the diversity of macrophages. In summary, even under homeostasis, macrophage phenotypes vary according to their location, developmental origin, local microenvironmental conditions, and genetic and epigenetic noise. This underlying complexity must be considered when trying to understand the variability of macrophages in pathological conditions. While most (but not all) TAMs have a hematopoietic origin (Murray, 2017; Qian and Pollard, 2010), macrophages are an intrinsically heterogeneous population. Keeping this in mind might help us to understand the diversity of their responses to inflammation and other signals.

## The inflammatory axis of macrophage polarization

When challenged, tissue-resident and circulating monocyte-derived macrophages alter their basal states in a process known as activation or ‘polarization’. The phenotypes of activated macrophages are often divided between two broad categories: M1 and M2. In response to inflammatory signals, such as interferon gamma (IFNγ) and lipopolysaccharides (LPSs; Box 1), macrophages polarize into the classical, or ‘M1’, state, which is characterized by specific changes in their transcriptional, morphological and secretory profiles (Mantovani et al., 2017; Murray, 2017; Murray et al., 2014). Gene expression changes include the upregulation of *Nos2* [which encodes the enzyme inducible nitric oxide synthase (iNOS)], and the secretion of pro-inflammatory signals, such as interleukin 6 (IL6) and IL12 (Murray et al., 2014). By contrast, alternatively activated macrophages (known as AAMs or as M2 macrophages) are polarized by anti-inflammatory signals, such as IL4 and IL13 (Mantovani et al., 2017; Murray et al., 2014), and upregulate genes, such as *Arg1*, *Mrc1* and *Cd163*. Although this binary distinction of polarized macrophages can be useful, it fails to account for the vast diversity of macrophage phenotypes (Lawrence and Natoli, 2011; Xue et al., 2014). As a result, multiple macrophage subcategories have emerged in recent years through a combination of multiple factors (Mantovani et al., 2017; Murray et al., 2014).

How do TAMs fit into this complex tapestry of phenotypes? For the most part, the intrinsic variabilities of macrophages from different locations are unfortunately ignored in the current system of macrophage categorization. In TAMs, the expression of genes normally associated with M2 macrophages, such *Arg1*, *Mrc1* and others, led to the likening of these two macrophage populations (Murray, 2018). This idea was further supported by the anti-inflammatory role that TAMs can acquire in tumors, where they have been shown to secrete pro-tumoral signals (Kitamura et al., 2015; Quail et al., 2016), recruit other anti-inflammatory cells (Curiel et al., 2004), de-differentiate into and from myeloid-derived suppressor cells (MDSCs; Box 1) (Corzo et al., 2010), and dampen the T cell response (Dong et al., 2002; Gallina et al., 2006; Rodriguez et al., 2004). As with TAMs, M2-like macrophages favor tumor growth (see, for example, Hughes et al., 2015; Lujambio et al., 2013; Murray, 2018). Consistently, the repolarization of TAMs into phenotypes that more closely resemble M1 macrophages has successfully produced anti-tumoral responses in pre-clinical murine models (Hughes et al., 2015; Mantovani et al., 2017; Pyonteck et al., 2013).

While there are clear similarities between some TAMs and stereotypical M2 macrophages, there are also some important differences. For example, transcriptional profiling of macrophages that reside in tumors in a murine model of spontaneous breast cancer (MMTV-PyMT) has shown that these TAMs represent a distinct population of myeloid cells; this subpopulation was almost absent before the onset of the disease but increased with tumor progression (Franklin et al., 2014). Using microarrays, the authors showed that this macrophage subpopulation had a different transcriptional profile to AAMs (or to M2 macrophages) and emerged in response to Notch (and not to Stat6) signaling, which transduces the response to IL4 and IL13 (Takeda et al., 1996) to induce M2 macrophages. Perhaps more importantly, TAMs display a variety of morphologies, uneven spatial distributions (Carmona-Fontaine et al., 2013; Joyce and Fearon, 2015; Wyckoff et al., 2007, 2011), variable expression of immunophenotyping proteins and different signal secretion profiles (Akkari et al., 2016; Franklin et al., 2014; Mantovani et al., 2017; Qian and Pollard, 2010; Quail et al., 2016). In addition, within tumors there is a combination of inflammatory and anti-inflammatory signals, such as TNF and IL13, that makes the phenotypic polarization of TAMs a dynamic process (Kratochvill et al., 2015).

Our definition of TAMs is strongly influenced by flow cytometry and by bulk genetic approaches, such as population RNA sequencing. Although flow cytometry provides rich data, it requires the destruction of tissue architecture and disregards spatial organization. Recently, microscopy has emerged as a powerful tool that can complement our molecular characterization of immune cells (Broz et al., 2014; Carmona-Fontaine et al., 2013, 2017; Gerner et al., 2012; Halle et al., 2016; Mukherjee et al., 2017). Using this approach, our group has recently shown that TAMs express M2 macrophages markers, such as *Arg1* and *Mrc1*, but they do so in distinct, nonoverlapping subpopulations (Carmona-Fontaine et al., 2017). The RNA sequencing of bulk TAM populations in this system would have mixed these two subpopulations together, thus giving the misleading impression that these two markers were co-expressed by TAMs. While taking tissue architecture into account is crucial, recent technological advances, such as single-cell sequencing, are also helping to uncover the full phenotypic diversity of TAMs and other cells (Lavin et al., 2014, 2017). A surprising observation from our microscopy-based analysis of TAMs in a spontaneous murine breast tumor model, was that *Arg1*-expressing TAMs were almost exclusively located within ischemic tumor regions, while *Mrc1*-expressing TAMs were found in perivascular and other well-nurtured tumor regions (Carmona-Fontaine et al., 2017). A perivascular *MRC1*-expressing TAM subpopulation in patients with breast cancer has been shown to be crucial for tumor relapse after chemotherapy, suggesting that the spatial patterns we observed in murine models might be conserved in human tumors (Hughes et al., 2015). Reinforcing the differences between TAMs, and macrophages polarized by anti/pro-inflammatory cues, ischemic TAMs co-express *Arg1* and *Nos2*, stereotypical M2 and M1 markers, respectively. The correlation between the proximity to the vasculature (and thus to oxygen and nutrient levels) and the distribution of TAM subpopulations, has prompted us and other researchers to investigate how local cell density, accessibility to blood vessels, and proximity to the tumor edge affect and diversify TAM phenotypes. The resulting evidence to date suggests that the range of metabolic microenvironments that emerge in tumors acts as an unrecognized axis of macrophage polarization with effects that are distinct from those of the inflammatory axis.

## The metabolic axis of macrophage polarization

**Fig. 2.**
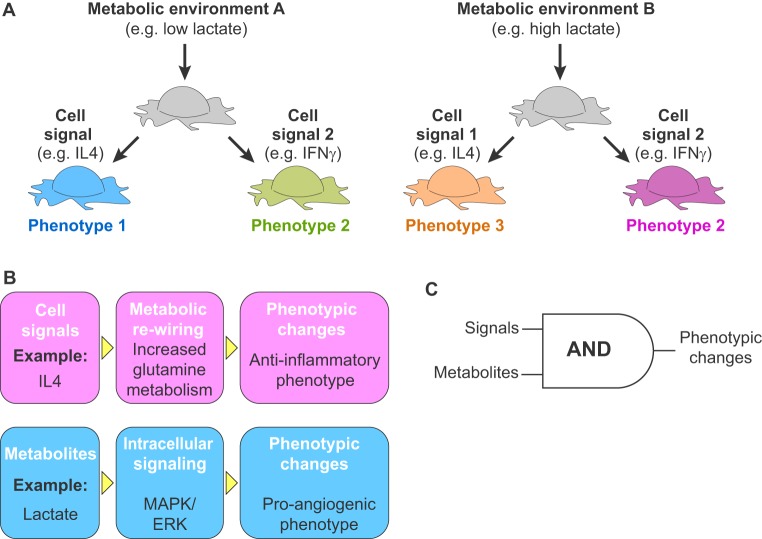
**Integration of metabolic and inflammatory signals.** (A) Schematic to show how the local metabolic microenvironment of a cell (gray) can modulate how its signals, such as by producing pro- and anti-inflammatory chemokines [such as interleukin 4 (IL4) and IFNγ, respectively], to alter cell phenotypes. (B) Cell signaling molecules, such as IL4 (top) are known to re-wire intracellular metabolism and to direct phenotypic change. Here, we highlight that extracellular metabolites (such as lactate, bottom) can act as cues that trigger intracellular signals and direct phenotypic changes. (C) The combined action of cell signaling and metabolites expands the repertoire of cellular responses. Here, we depict these two sources of cell modulation as part of an AND gate.

Metabolic rewiring is an integral aspect of how macrophages respond to external signals (Ganeshan and Chawla, 2014; O'Neill and Hardie, 2013). For example, strong inflammatory stimuli provided by a combination of IFNγ and LPS in murine bone marrow-derived macrophages lead to an accelerated glycolytic metabolism (Tannahill et al., 2013) and to a ‘broken’ version of the tri-carboxylic acid cycle (TCA), which leads to an accumulation of malate and itaconate (Jha et al., 2015; Lampropoulou et al., 2016). Conversely, murine bone marrow-derived macrophages exhibit enhanced oxidative phosphorylation (Vats et al., 2006) and increased glutamine metabolism (Jha et al., 2015) when treated with anti-inflammatory signals, such as IL4. Consistent with this evidence, IL10 – another anti-inflammatory signal – inhibits glycolysis and promotes oxidative phosphorylation, possibly via the inhibition of mTOR signaling (Ip et al., 2017). How macrophage metabolism changes in response to chemokines and to other cell signals has been extensively reviewed elsewhere (Ganeshan and Chawla, 2014; O'Neill and Hardie, 2013). However, we know much less about how extracellular metabolites act as signals and alter intracellular signaling, cell metabolism and macrophage function. Here, we will focus on how the availability of oxygen, nutrients and other extracellular metabolites, interferes with and modulates other types of cell signals to directly change macrophage phenotypes (Fig. 2).

Monocyte-derived TAMs are known to migrate towards hypoxic, avascular regions of multiple tumor types (Cramer et al., 2003; Murdoch et al., 2004). The stabilization of hypoxia-inducible factor 1-alpha (HIF-1α) by low oxygen levels can activate inflammatory programs in macrophages via the recruitment of components of the inflammasome (Box 1) (Wen et al., 2012). HIF-1α activation can also be driven directly by inflammatory signals [such as LPS (Nishi et al., 2008; Tannahill et al., 2013)] and by pseudohypoxia, when the accumulation of metabolites, such as succinate (Chouchani et al., 2014; Tannahill et al., 2013) or low pH (Intlekofer et al., 2017), stabilize HIF-1α even under normal oxygen tension. Thus, direct (via low oxygen) or indirect hypoxia (via the activation of HIF-1α independently of oxygen levels) can trigger an inflammatory program in macrophages in the absence of pro-inflammatory chemokines.

Excessive ischemia in avascular tumor regions can lead to the accumulation of lactic acid, which lowers the extracellular pH to levels that are sustainable for some tumor cells but lethal for TAMs (Carmona-Fontaine et al., 2013). A combination of recruitment by chemokines, such as CCL2 (Shi et al., 2011a), attraction towards hypoxia (Cramer et al., 2003; Murdoch et al., 2004) and death by low pH (Carmona-Fontaine et al., 2013), has been proposed to explain the complex distribution patterns of TAMs observed in murine models (Carmona-Fontaine et al., 2013; Gocheva et al., 2010; Wyckoff et al., 2007). Sublethal levels of lactic acid can have interesting effects on macrophages. Recent evidence shows that bone marrow-derived macrophages (BMDMs) and TAMs treated with lactate express some of the genes associated with anti-inflammatory macrophages (Colegio et al., 2014). This effect was not caused by pH changes, but mediated directly by lactate in an HIF-1α-dependent manner (Colegio et al., 2014).

**Fig. 3.**
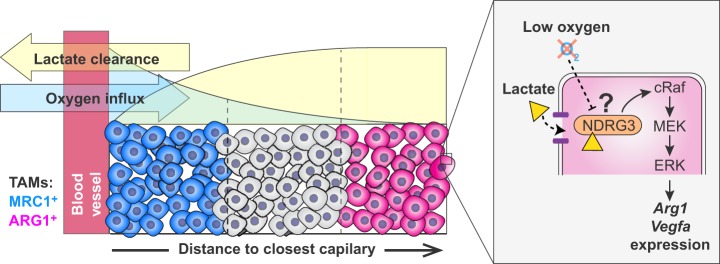
**Effect of ischemia on TAMs.** Schematic of a cross-section through a tumor and its associated blood vessel, with blue representing well-perfused tumor cells, red representing ischemic cells and gray representing cells at intermediate locations. Lactate levels (yellow) increase with distance from the blood vessel, whereas oxygen levels (light blue) decrease with distance from the blood vessel. Lactate combined with hypoxia act synergistically to activate gene expression in TAMs, which are present within the gradient of ischemia. A putative mechanism by which this could occur is shown in the inset box (right) and involves NDRG3, a lactate-dependent hypoxia sensor that signals via the MAPK/ERK pathway to activate the expression of the TAM markers, including *Arg1*, *Mrc1* and *Vegfa*.

Ischemic environments feature a combination of hypoxia, low nutrient levels and the accumulation of waste products, such as lactate. Many studies have isolated single ischemic variables – for example, hypoxia – and have examined their effects on cellular biology. This approach has helped to establish how cells respond to specific metabolic cues. But because cells do not sense these cues in isolation, we might be missing important information about how these metabolic cues interact or synergize to change cell fate. To overcome this, our group recently developed an *ex vivo* system to study the effect of ischemia on cells, including macrophages (see ‘Perspective: the need for tools to study the metabolic microenvironment’ section). Using this system, we have shown that the general macrophage response to ischemia is primarily driven by the combined effect of lactate and hypoxia (Fig. 3). This combination directly activates MAPK/ERK signaling via cRaf (also known as RAF1), which in turn triggers a transcriptional profile that is quite distinct from typical ‘M1’ and ‘M2’ macrophages (Carmona-Fontaine et al., 2017). An interesting possibility is that macrophages and TAMs integrate these two metabolic cues via NDRG family member 3 (NDRG3), which has been shown to be a lactate-dependent hypoxia sensor that signals via cRaf (Fig. 3) (Lee et al., 2015). While this pathway has so far not been shown to operate in macrophages or in other immune cells, NDRG3 could represent an interesting target for therapy and the modulation of the tumor microenvironment. JAK-STAT signaling is an essential part of the pro-inflammatory (via STAT1) and anti-inflammatory (via STAT6) response that generates ‘M1’ and ‘M2’ macrophages, respectively (Murray, 2007). However, JAK-STAT signaling is dispensable for the macrophage response to ischemia, reinforcing the idea that this response to metabolic cues is distinct to the inflammatory axis of polarization (Carmona-Fontaine et al., 2017). It is also noteworthy that the activation of HIF-1α is not sufficient to recapitulate the macrophage response to ischemia [although HIF-1α might still be required for other aspects of macrophage adaptation to hypoxic conditions (Colegio et al., 2014)], emphasizing the relevance of studying how different metabolic cues act in concert.

**Fig. 4.**
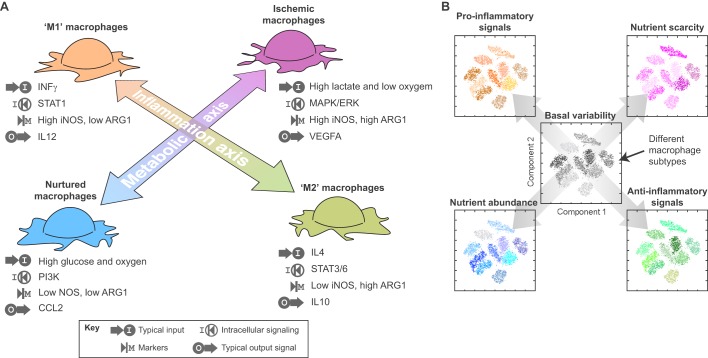
**The metabolic axis of macrophage polarization.** (A) A proposed metabolic axis of macrophage polarization that is orthogonal to (but still able to interact with) the traditional inflammatory axis that leads to M1/M2 macrophage polarization. Typical input signals are shown (such as oxygen, glucose, lactate and cytokine levels), as well as intracellular mediators, molecular markers used to distinguish them and secreted chemokines for these differently polarized examples. These signals and markers are provided as examples rather than reflecting a complete view of these different phenotypes. (B) A conceptual representation of how the underlying phenotypic heterogeneity of a cell population (such as TAMs) is modulated by inflammatory or metabolic perturbations. In this representation, the basal heterogeneity of the cell population is shown at the center as a two-dimensional landscape that is commonly used to represent heterogeneous multidimensional data (e.g. as in PCA and tSNE plots). In these types of plots, subpopulations with common features cluster together. After perturbations, all subpopulations change, and we depict this with similar plots, but now the subpopulations have shifted to a different state (represented with different colors). For example, under nutrient deprivation, all subpopulations change (represented as a shift from gray to magenta hues) but they somehow maintain their original diversity as they remain in separate clusters. When analyzing real data, however, this might change as some perturbations can either unify, diversify further, or reconfigure the original phenotypic diversity, affecting the shape and size of the clusters in the plot.

The transcriptional response to ischemia also differs from the macrophage response to inflammatory signals. For example, the quintessential ‘M1’ marker is the expression of *Nos2* (that encodes iNOS), while murine ‘M2’ macrophages express high *Arg1* levels (Murray et al., 2014) (Fig. 4). In contrast, ischemic macrophages co-express both of these markers and the combination of hypoxia and high lactate inhibits the expression of the ‘M2’ marker *Mrc1* (Carmona-Fontaine et al., 2017). These data suggest that the metabolic polarization of macrophages might lie outside of the M1/M2 axis of macrophage activation. These findings also help to explain our observation that *Arg1* and *Mrc1* are expressed in mutually exclusive TAMs subpopulations in a murine model of breast cancer (Carmona-Fontaine et al., 2017). Interestingly, Toll-like receptors can signal via the ERK pathway and trigger a macrophage response that resembles the response to intracellular pathogens (El Kasmi et al., 2008). *Arg1* levels can also be regulated by a Stat6-independent autocrine signaling mechanism that is mediated by CCAAT/enhancer-binding protein beta (C/EBPβ) (Qualls et al., 2010). Emerging evidence in the literature has shown that *Arg1* is also upregulated in other pathological ischemic conditions, such as in granulomas in tuberculosis (Duque-Correa et al., 2014; Mattila et al., 2013) and during retinal vein occlusion (Martin et al., 2018; Narayanan et al., 2013), arguing that the metabolic axis of macrophage polarization is not exclusive to tumors but a general aspect of macrophage biology. In summary, in response to ischemia, macrophages adopt a polarized state, mediated by the MAPK/ERK pathway, that is different to ‘M1’ and ‘M2’ polarization.

Macrophages also face ischemia in noncancerous contexts as part of their physiological roles. For example, when a nerve is severed, neighboring capillaries are also usually damaged, which creates a hypoxic region near the wound (Cattin et al., 2015). Macrophages respond directly to hypoxia by migrating to the wound and attracting endothelial cells via the secretion of vascular endothelial growth factor (VEGF) proteins. These recruited endothelial cells stimulate the revascularization of the severed region, which is a required step in the nerve repair process (Cattin et al., 2015). It is possible that tumor ischemia triggers a similar mechanism in TAMs, and thus part of the behavior of metabolically polarized macrophages can be co-opted from physiological wound healing roles (Schäfer and Werner, 2008; Weber et al., 2016). Consistent with this idea, TAMs within ischemic tumor regions express VEGF subtypes and attract endothelial cells, likely to re-vascularize these regions (Carmona-Fontaine et al., 2017; Lewis et al., 2000; Murdoch et al., 2004). It has been proposed that deranged tumor vascularization and leaky blood vessels facilitate tumor metastasis (Mazzone et al., 2009), and that part of that leakiness might come from excessive re-vascularization (Wenes et al., 2016). This group has recently shown that when the angiogenic response of macrophages is diminished, tumors create better-formed and functional vessels, concomitant with a reduction in metastasis. They achieved this by increasing the glucose uptake and glycolytic rate of TAMs via the inhibition of REDD-1, a negative regulator of the mTOR pathway, in murine tumors (Wenes et al., 2016). It is well known that hypoxia directly triggers the expression of VEGF proteins (Fukumura et al., 1998; Shweiki et al., 1992), and a similar effect has been reported for lactate (Colegio et al., 2014). However, in mouse bone marrow-derived macrophages, the combination of lactate and hypoxia produced a synergistic effect on *Vegfa* expression, and consequently on VEGFA secretion, which largely exceeded the effect of lactate or hypoxia alone (Carmona-Fontaine et al., 2017). The synergy between these signals further supports the idea that macrophages integrate the levels of multiple metabolites to gain more robust information about their environments.

The levels of many other nutrients and metabolic waste products are also likely to alter the phenotype of TAMs. For example, amino acids are heterogeneously distributed within tumors in which low glutamine levels are found in blood-deprived tumor regions (Pan et al., 2016). This local glutamine deprivation leads to histone hypermethylation and to the dedifferentiation of cancer cells (Pan et al., 2016). It would therefore be interesting to study whether glutamine levels produce similar epigenetic changes in TAMs. Macrophages rely on glucose for ATP production and as a carbon source (Cramer et al., 2003; Kelly and O'Neill, 2015). Glucose is also vital for a pro-inflammatory response in macrophages, and the inhibition of glucose metabolism causes a diminished inflammatory response in macrophages (Haschemi et al., 2012; Tan et al., 2015). Consistent with this, the stable overexpression of the glucose transporter GLUT1 (also known as SLC2A1) has been reported to place macrophages in a hyperinflammatory state (Freemerman et al., 2014). Thus, glucose levels might contribute to how macrophages respond to ischemia and blood perfusion. Considering these data, it is not surprising that the activity and levels of nutrient sensors, such as mTORC1 and AMPK, also affect macrophage function, as has been extensively reviewed elsewhere (Biswas, 2015; Covarrubias et al., 2015; Hardie, 2015). mTORC1 is implicated in autophagy and the synthesis of lipids, proteins and nucleotides, along with metabolic reprogramming and differentiation in macrophages (Covarrubias et al., 2015; Weichhart et al., 2015). In macrophages and other mammalian cells, AMPK (also known as PRKAA2) is associated with cellular metabolic reprogramming in response to nutrient deprivation (Hardie, 2015) and influences macrophage polarization (Sag et al., 2008). Overall, there is considerable evidence that macrophages sense their metabolic microenvironment and modulate their functional immunophenotypes according to this information. While efforts have been made to classify the macrophage response to extracellular organisms along the M1 and M2 axis, we believe that their metabolic responses are orthogonal and distinct from the responses to the inflammatory axis of macrophage polarization. Thus, we propose that the degree of blood perfusion and ischemia in the microenvironment establishes a ‘metabolic axis’ of macrophage polarization. As we discuss in the next section, similar metabolic cues can affect other immune cells and thus this metabolic axis could affect the functional polarization of other immune cells.

## Metabolic polarization of other immune cells

More than 60 years ago, the pioneer in organ transplantation, Peter Medawar, asked how embryos avoid rejection (Billingham et al., 1953). The answer came ∼40 years later, along with the first evidence of metabolites that affect the immune system directly. Indoleamine-2,3-dioxygenase (IDO) functions as a rate-limiting-step enzyme in tryptophan catabolism. Cells that express high levels of IDO rapidly deplete extracellular tryptophan, which diminishes T-cell proliferation and activity (Munn et al., 1999). *Ido* (also known as *Ido1*) is expressed in early mammalian embryonic structures, and tryptophan levels drop during pregnancy (Schrocksnadel et al., 1996). This led Munn and colleagues to hypothesize that tryptophan metabolism creates an immunosuppressive environment that protects the embryo from the maternal immune system (Munn et al., 1998). The immunomodulatory effect of tryptophan metabolism could in theory also be relevant for immune evasion in tumors (Sheridan, 2015). Cancer-associated fibroblasts (CAFs), macrophages and other antigen-presenting cells (APCs), and sometimes tumor cells themselves, upregulate IDO, exacerbating tryptophan catabolism, to create an immunosuppressive tumor microenvironment that prevents T-cell activation by inducing anergy (Box 1) and apoptosis (Moffett and Namboodiri, 2003; Platten et al., 2012). This immunosuppressive effect occurs via at least two mechanisms: direct depletion of tryptophan, which fuels biomass production during the rapid expansion of activated T cells; and the accumulation of immunosuppressive metabolites derived from tryptophan catabolism, such as kynurenine, 3-hydroxyanthranilate and quinolinate (Moffett and Namboodiri, 2003; Platten et al., 2012). These tryptophan-derived catabolites appear to have additional immunomodulatory effects; for example, they can act as ligands of the aryl hydrocarbon receptor (AHR). Endogenously produced metabolites, including kynurenine, directly activate the AHR (Opitz et al., 2011). Kynurenine-dependent activation of AHR has been shown to skew the differentiation of naive T cells towards FoxP3^+^ regulatory T cells (Tregs; Box 1) cells rather than to T_h_17 cells (Box 1) (Mezrich et al., 2010; Nguyen et al., 2010), thus promoting immunosuppressive features in T cells. A careful look at *Ido1/2* double-knockout mice could also illuminate the role of these enzymes in regulating the immune response. The immunomodulatory effects of IDO and tryptophan metabolism are still under active investigation, and both are promising targets for new immunotherapy drugs (Sheridan, 2015).

Low oxygen tensions and high levels of lactate appear to both independently and synergistically suppress adaptive immunity and to augment innate immune system function through a variety of mechanisms that target inflammation. In the innate immune system, hypoxia increases the survival of neutrophils and delays apoptosis (Carmeliet and Jain, 2011). HIF-1α has also been shown to considerably increase the release of pro-inflammatory cytokines and pro-stimulatory molecules by dendritic cells (Kumar and Gabrilovich, 2014; Nizet and Johnson, 2009). In the adaptive immune system, HIF-1α has a variety of functions (Eltzschig and Carmeliet, 2011), including driving the expression of inhibitory receptors by activated T cells (Freemerman et al., 2014), increasing the number of Tregs by modulating FoxP3 levels (Ben-Shoshan et al., 2008), and inducing a shift from a type 1 helper T cell to a type 2 helper T cell (Box 1), which in turn increases the production of IL10 and decreases IFNγ levels (Ben-Shoshan et al., 2009). The cytotoxic abilities of cytotoxic T lymphocytes (CTL; Box 1) and natural killer (NK; Box 1) cells become inhibited with increased levels of lactate. With high levels of lactate, there is also a decrease in CTL proliferation (Fischer et al., 2007), a reduction in the ability of NK and T cells to surveil for the presence of tumors (Brand et al., 2016; Rizwan et al., 2013; Xie et al., 2014), and a reduction in T cell mobility (Haas et al., 2015).

Ischemia is also associated with an increase in the activity of lactate dehydrogenase (LDH) and malate dehydrogenase (MDH), which use alpha-ketoglutarate as a substrate to make (L)-2-hydroxyglutarate (L-2HG) (Intlekofer et al., 2015, 2017). This metabolite is the stereoisomer of (R)-2-hydroxyglutarate (R-2HG), which is produced by tumorigenic mutations in isocitrate dehydrogenase (IDH) (Dang et al., 2009; Ward et al., 2010; Xu et al., 2011). Both isoforms of 2HG can have profound effects on cell phenotypes and differentiation, as they can directly affect histone modifications; they can thus play a role in the balance between undifferentiated and differentiated cell states (Lu et al., 2012). In T cells, L-2HG has dramatic effects on T cell activation, almost wholly abolishing T-cell activation and promoting instead a regulatory T-cell differentiation program (Tyrakis et al., 2016).

Metabolites are primarily the energy and biomass resources that are required for cell function. The modulation of glucose (Jha et al., 2015), phosphoenolpyruvate (Ho et al., 2015) and potassium (Eil et al., 2016) levels have been shown to affect immune response or to shift immune cell phenotypes towards a more immunosuppressive state (Ghaderian et al., 2010; Ghesquiere et al., 2014). Succinate can act as a chemokine that enhances chemotaxis in dendritic cells (Rubic et al., 2008). Glucose availability also modulates immune cell phenotypes. Limiting glucose levels during T-cell activation can prevent effector T-cell expansion (Buck et al., 2017; Chang et al., 2015; Frauwirth et al., 2002; Wang et al., 2011) and IFNγ production (Cham and Gajewski, 2005). It has also been shown that tumor cells compete with T cells for glucose, and often T cells are suppressed as a result of this competition (Chang et al., 2015). The activation of dendritic cells also depends on glucose levels (Everts et al., 2014; Lawless et al., 2017; Thwe et al., 2017). Intriguingly, recent evidence suggests that the effect of glucose in dendritic cells depends on the type – and activation state – of neighboring cells. For example, glucose can facilitate inflammatory functions in dendritic cells (Everts et al., 2014), but this effect is inhibited in the presence of inflammatory T cells (Lawless et al., 2017). The reason for this is that the high glycolytic rate of inflammatory T cells causes glucose to become locally deprived, which hampers the inflammatory response of dendritic cells (Lawless et al., 2017). This is another example of how the spatial structure of the tissue, and the relative position of different cells, impact cell phenotypes and behavior.

Nutrients and metabolites are essential for biological organization. Given their crucial role, all life forms have refined mechanisms for sensing and adapting to extracellular metabolite levels (Chantranupong et al., 2015; Palm and Thompson, 2017). Without trying to create a complete and exhaustive list of how every metabolite affects all immune cells, we have shown a few examples of how metabolite sensing is at the core of immune cell behavior and functions. Although this feature is not exclusive to tumor-infiltrated immune cells, nutrient sensing and adaptation might be particularly important within the deranged metabolic microenvironment found in most tumors.

## Perspective: the need for tools to study the metabolic microenvironment

Our understanding of how cellular phenotypes change in response to the local metabolic microenvironment is limited by two main challenges. The first challenge is the need to measure cell phenotypes, within their spatial context, ideally at single-cell resolution. As we mentioned before, single-cell sequencing is redefining how we look at cell populations *in vivo* (Lavin et al., 2014, 2017). Although this is a fantastic development, it still requires the destruction of the tissue architecture and the loss of important spatial information. In addition, sample preparation is relatively slow, especially relative to the time scales at which cellular metabolism changes. A complementary approach is cell cytometry, where fixed or live cells can be examined using fluorescent dyes, antibodies and genetically-encoded probes (Broz et al., 2014; Carmona-Fontaine et al., 2013, 2017; Gerner et al., 2012; Halle et al., 2016; Mukherjee et al., 2017). We have worked significantly on this approach, but the limited number of fluorophores available to detect and discriminate between cells limits the range of parameters one can analyze simultaneously. The matrix-assisted laser desorption ionization (MALDI) imaging of antibodies labeled with metals, which can then be analyzed with mass spectrometry, might help to resolve this issue (Angelo et al., 2014), but this approach requires sample destruction and it is not widely available.

The second major challenge is to measure, without perturbing, the temporal and spatial distribution of metabolites in tissues. MALDI-based metabolomics is one alternative but retains some of the same issues we described above (Chughtai and Heeren, 2010). Genetically encoded fluorescent probes have also recently been developed that report levels of specific metabolites. For example, the probe Laconic can be used to determine intracellular lactate levels with high accuracy (San Martín et al., 2013). Measuring extracellular metabolite levels is more challenging, partly because of the fine spatial resolution required to study them *in vivo.*

*In vivo* studies of metabolites are complex, and it is often difficult to determine whether metabolites are directly responsible for the observed phenotypic changes. A potentially transformative tool would be a method that could be used to alter the local levels of specific metabolites, at will. At the same time, conventional *in vitro* culture systems lack the spatial structure that provides heterogeneity in metabolic microenvironments. To overcome this, we have developed the metabolic microenvironment chamber (MEMIC), in which cultured cells spontaneously create gradients of ischemia (Carmona-Fontaine et al., 2013, 2017). This tool supports the detailed analysis of how gradients of metabolites affect tumor and immune cells *ex vivo*. However, the MEMIC is not without its limitations. For example, in order to generate the gradients, cells are enclosed in a small volume in glass, which hampers access to the cells to perform biochemical analyses. The small dimensions of the MEMIC also complicate certain analyses, such as RNA-sequencing or metabolomics, because of small sample yields. A system similar to the MEMIC, but one that supports better cellular yield and sample accessibility, is urgently required. Our own, and many other groups, are working to develop these and other techniques that could be used to understand and modulate how metabolites alter cell function. By combining these techniques with *in vivo* observations, manipulations and measurement of local metabolite levels, we hope to be able to better understand, and potentially to control, the cellular adaptation to different tumor metabolic microenvironments.

This article is part of a special subject collection ‘Cancer Metabolism: models, mechanisms and targets’, which was launched in a dedicated issue guest edited by Almut Schulze and Mariia Yuneva. See related articles in this collection at http://dmm.biologists.org/collection/cancermetabolism.
